# A versatile high-average-power ultrafast infrared driver tailored for high-harmonic generation and vibrational spectroscopy

**DOI:** 10.1038/s41598-023-46325-3

**Published:** 2023-11-01

**Authors:** Nicolas Thiré, Gourab Chatterjee, Yoann Pertot, Olivier Albert, Gabriel Karras, Yu Zhang, Adam S. Wyatt, Michael Towrie, Emma Springate, Gregory M. Greetham, Nicolas Forget

**Affiliations:** 1 Fastlite, 165 route des cistes, 06600 Antibes, France; 2 STFC Central Laser Facility, Harwell Science and Innovation Campus, Didcot, Oxfordshire OX11 0QX, UK; 3 Present Address: SLAC National Accelerator Laboratory, 2575 Sand Hill Road, Menlo Park, CA 94025 USA; 4 Present Address: Diamond Light Source, Harwell Science and Innovation Campus, Didcot, Oxfordshire OX11 0DE UK; 5 Present Address: CNRS UMR7010 INPHYNI, 1361 route des Lucioles, 06560 Valbonne, France

**Keywords:** Ultrafast lasers, High-harmonic generation, Infrared spectroscopy, Raman spectroscopy

## Abstract

We report on an ultrafast infrared optical parametric chirped-pulse amplifier (OPCPA), pumped by a 200-W thin-disk Yb-based regenerative amplifier at a repetition rate of 100 kHz. The OPCPA is tunable in the spectral range 1.4–3.9 $\upmu$m, generating up to 23 W of < 100-fs signal and 13 W of < 200-fs idler pulses for infrared spectroscopy, with additional spectral filtering capabilities for Raman spectroscopy. The OPCPA can also yield 19 W of 49-fs 1.75-$\upmu$m signal or 5 W of 62-fs 2.8-$\upmu$m idler pulses with active carrier-to-envelope-phase (CEP) stabilisation for high-harmonic generation (HHG). We illustrate the versatility of the laser design, catering to various experimental requirements for probing ultrafast science.

## Introduction

High-repetition-rate high-average-power thin-disk, slab and fibre-based lasers are gaining popularity, owing to their robust compact design, excellent beam quality and reliable power stability^1–8^. Whilst providing adequate peak powers to trigger various non-linear processes of interest, these laser sources offer an improved signal-to-noise ratio and fast data acquisition time-scales. This is vital in combating space-charge effects in condensed-phase photoemission processes, improving low-yield coincidence measurements, or in experiments with naturally degradable samples^9–17^.

**Figure 1:**
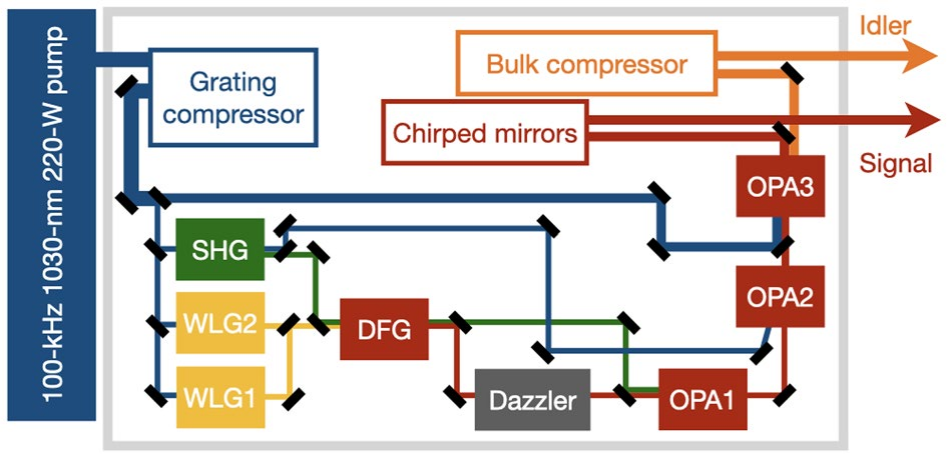
OPCPA design. Following compression, 150 W of the pump drives OPA3. The remaining $\sim$ 50 W is frequency-doubled (SHG) and the 515-nm output pumps the DFG and OPA1 stages, whereas the 1030-nm residual pump drives OPA2. The DFG is seeded by the white-light-generation stage – either WLG1 or WLG2, depending on the passive CEP stability requirement (further details in the manuscript). The idler is propagated, via pulse-shaping in a Dazzler, to seed the subsequent OPAs.The signal and the idler are compressed via chirped mirrors and bulk compressors, respectively. The OPCPA also includes f–2f interferometers for active CEP stabilisation using the Dazzler, as well as environmental and shot-to-shot jitter monitoring diagnostics (not shown in the figure).

Here, we illustrate the performance of an ultrafast infrared OPCPA (TwinStarzz from Fastlite), driven by a 200-W pump source (Dira from Trumpf Scientific), operating at a repetition rate of 100 kHz. The OPCPA design is devised to satisfy a host of criteria for a suite of ultrafast spectroscopic techniques, ranging from HHG-based transient angle-resolved photoemission spectroscopy (tr-ARPES) and x-ray absorption spectroscopy (tr-XAS) to time-resolved infrared and Raman spectroscopy. In tr-APRES with solid-state samples, the number of photoelectrons emitted per pulse is constrained by space charge effects, which limits the energy of the driving pulse and affects data statistics. A higher repetition-rate laser is therefore beneficial. Similarly, in tr-XAS, accessing elemental x-ray absorption edges profits from a mid-infrared wavelength for the HHG driver^18^. However, the process suffers from a low conversion efficiency and is therefore aided by an improved average HHG photon flux at high repetition rates. Vibrational spectroscopy often demands spectral tunability of the laser to selectively excite molecular vibrations. In this regard, the appeal of an OPCPA design is the possibility to adjust the laser spectral bandwidth, without resorting to complex pulse stretching and compression, to suit the purpose of the experiment. Whilst broad spectral coverage permits accessing extended spectroscopic transitions in absorption spectroscopy, it limits energy resolution in frequency-resolved techniques – for example, in Raman spectroscopy. On the other hand, the ability to reduce spectral bandwidth can increase spectral brightness by concentrating laser energy into ‘useful’ bandwidths when broad spectral coverage is not required.

In order to satisfy the diverse experimental requirements, the OPCPA is designed to operate interchangeably amongst (1) a tunable infrared spectroscopy mode, (2) a tunable Raman spectroscopy mode and (3-4) two fixed-wavelength actively CEP-stabilised HHG modes. This functional and flexible, multifaceted design constitutes the salient feature of the OPCPA architecture.

## Results and discussion

The seed-source laser is a passively mode-locked fibre-based oscillator and pre-amplifier (OneFive), which seeds the Yb:doped thin-disk regenerative amplifier (Dira). Operated at a repetition rate of 100 kHz, the amplifier produces an output power of 220 W with a prepulse intensity contrast of $10^{-3}$. The collimated amplifier output has a near-Gaussian beam profile with $M^2=1.1$ along both the major and minor axes, and a $1/e^2$ beam diameter of $\sim$ 4 mm. A two-pass dielectric-grating compressor (Fig. 1) with an overall efficiency of 90% yields a near-transform-limited pulse duration of 860 fs. Following compression, the thermally-induced pointing drift of the beam is compensated (up to $\sim$ 1 mrad) by active pointing stabilisation (MRC), monitored by quadrant photodiodes.

150 W of the 200-W output from the compressor is used to pump the final optical parametric amplifier (OPA) stage—OPA3 (Fig. 1). The remaining $\sim$ 50 W is frequency-doubled in a 1-mm thick antireflection-coated BBO crystal ($\theta =23.4^\circ , \phi =0^\circ$). The 5-W 515-nm output from the second-harmonic-generation (SHG) stage is used to pump the initial difference-frequency-generation (DFG) and OPA1 stages, whereas the residual $\sim$ 45 W of the 1030-nm pump is used to drive the penultimate OPA2 stage. A small fraction of the compressor output is used for white-light-generation (WLG1) in a 15-mm thick YAG crystal, which is continuously rastered at a speed of $\sim 1$ mm/min to avoid damage^19,20^. Whilst seeding with WLG1 provides passive CEP stability for the final idler output, an alternative frequency-doubled WLG2 stage is utilised to maintain passive CEP stability for the final signal output (further details later).

The white-light-seeded and 515-nm-pumped collinear DFG stage comprises an antireflection-coated 3.7-mm thick LBO crystal ($\theta =90^\circ , \phi =13.8^\circ$). The $\sim$ 0.5-W near-infrared signal is rejected, whereas the $\sim$ 0.2-W idler (1.4–1.85 $\upmu$m) is propagated to seed the subsequent OPA stages, following pulse-shaping in a lithium-niobate (LN) acousto-optic programmable dispersive filter (Dazzler, Fastlite). The pulse-shaping introduces linear and higher-order dispersion to optimise the temporal overlap of the seed with the pump in the subsequent OPA stages and allows fine-tuning of the final output compression. The $\sim$ 10% transmission efficiency of the Dazzler provides $\sim$ 20 mW of shaped seed to OPA1.

The collinear OPA1 stage comprises an antireflection-coated 2.8-mm thick LBO crystal ($\theta =90^\circ , \phi =13.8^\circ$). It is seeded by the pulse-shaped idler from the DFG and driven by the residual 515-nm pump from the DFG. OPA1 amplifies the seed to a $\sim$ 0.2-W signal (at 1.4-1.85 $\upmu$m), which then seeds OPA2, whereas the idler is rejected. The non-collinear OPA2 stage is based on an antireflection-coated 5-mol% Mg-doped periodically-poled fan-out LN crystal with a quasi-phase-matching period of 24-32 $\upmu$m^21^. It is pumped by the unconverted $\sim$ 45 W of 1030-nm light from the SHG stage. The 5-W signal from OPA2 seeds the final collinear OPA3 stage. OPA3 comprises an antireflection-coated 1.5-mm thick low-defect MgO:LN crystal ($\theta =45.4^\circ , \phi =30^\circ$) at a temperature of 120$^{\circ }$C and is pumped by 150 W of the 1030-nm output from the grating compressor^22^. The pump intensity in OPA2 and OPA3 are maintained at $\sim 70$ GW/cm$^2$.

**Figure 2:**
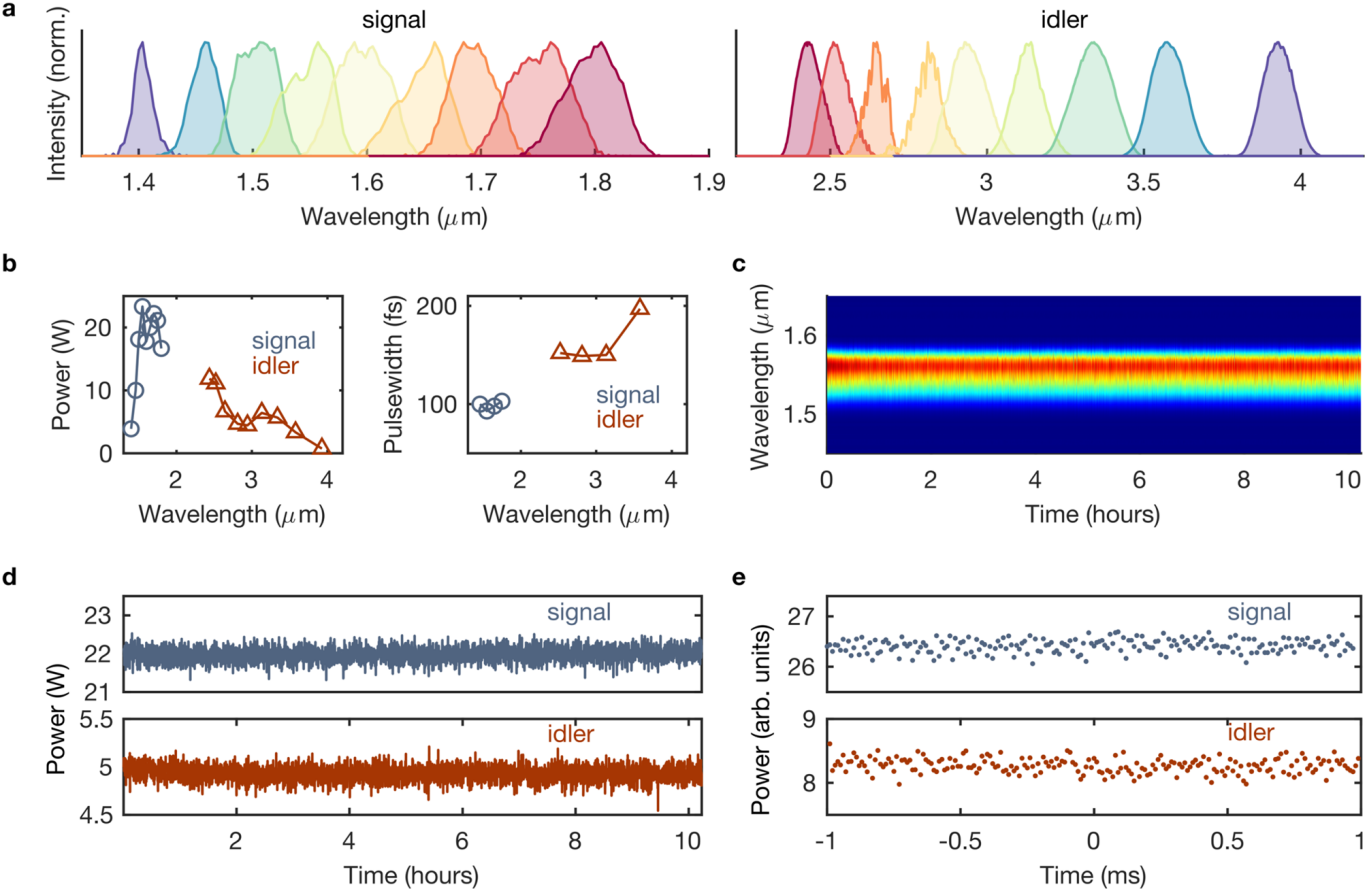
Tunable infrared spectroscopy mode. (**a**) Self-normalised signal and idler spectra. (**b**) Output power and measured pulse durations. (**c**) Spectral stability of the signal at 1.55 $\upmu$m. (**d**) Power stability of the signal at 1.55 $\upmu$m and the corresponding idler at 3.1 $\upmu$m, with rms fluctuations of 0.8% and 1.5%, respectively, over 10 h. (**e**) Shot-to-shot stability, with rms fluctuations of 0.5% and 1.4% for the signal and idler, respectively, over 2 ms.

The signal from OPA3 is compressed by two different sets of chirped mirrors in order to cover the entire 1.4–1.85 $\upmu$m spectral range of the signal. The corresponding idler is compressed by anti-reflection-coated bulk silicon windows of varying thicknesses and a pair of adjustable silicon wedges. The spectral tuning is performed completely with the help of motorised controls.

The OPCPA generates up to 23 W of < 100-fs signal ($\sim$ 50 fs at 1.75 $\upmu$m) and 13 W of < 200-fs idler, spanning the spectral ranges 1.4-1.8 $\upmu$m and 2.3-3.9$\upmu$m, respectively (Fig. 2). The spectra (Fig. 2a) are measured simultaneously with a 0.9–2.5 $\upmu$m spectrometer (NIRQuest, Ocean Optics) and a 1–5 $\upmu$m multi-octave infrared spectrum analyser (Mozza, Fastlite). The power dip at $\sim$ 3 $\upmu$m (Fig. 2b) can be attributed to absorption bands in both lithium niobate and the ambient water-vapour outside the OPCPA unit, which is purged with dry air. The pulse durations (Fig. 2b) are measured by an SHG-based frequency-resolved optical gating (FROG)^23^ device (Frozzer, Fastlite), coupled to a spectrometer (Avantes or NIRQuest). The spectral stability of the signal is also illustrated (Fig. 2c). The root-mean-squared (rms) power fluctuations, measured over a time period of 10 h with thermopile powermeters, are 0.8% for the 1.55-$\upmu$m signal and 1.5% for the corresponding 3.1-$\upmu$m idler (Fig. 2d). The OPCPA is equipped with an in-built fibre-coupled shot-to-shot energy monitor (Big Brozzer, Fastlite), which shows rms fluctuations of 0.5% and 1.4% for the signal and idler, respectively, measured over 0.2 ms (200 shots). This is independently corroborated by measuring the shot-to-shot noise of the signal and idler scatter with extended-InGaAs and MCT detectors (Fig. 2e). For comparison, the pump (Dira) shows an rms fluctuation of $<0.1$%, measured at full power (226 W) over 3 h, and a shot-to-shot rms fluctuation of 0.4% over $10^3$, $10^4$, and $10^5$ shots. The shot-to-shot stability is pivotal in discerning weak spectroscopic signals amidst the background noise – for instance, in two-dimensional infrared (2DIR) spectroscopy^24^.

**Figure 3:**
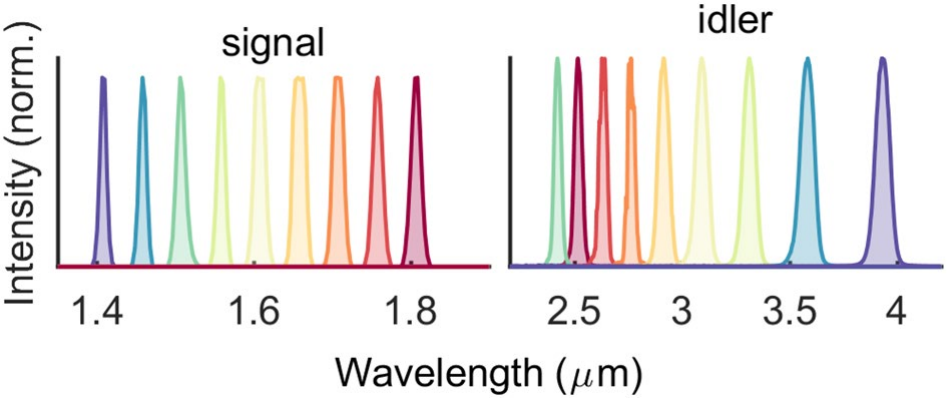
Tunable Raman spectroscopy mode. Self-normalised signal and idler spectra with up to 21 W and 10 W power, respectively, and $\sim$ 50 cm$^{-1}$ spectral bandwidth.

The Dazzler-based pulse-shaping facilitates convenient frequency filtering of the seed. Consequently, the broadband femtosecond pulses can readily be converted to narrow-band ($\sim$ 50 cm$^{-1}$) pulses with up to 21 W and 10 W of power for the signal and idler, respectively (Fig. 3). The increase in spectral brightness is of benefit in time-resolved Raman spectroscopy. Whilst a higher spectral resolution (typically $<20$ cm$^{-1}$) is preferable for condensed-phase Raman experiments, further spectral filtering of the seed leads to reduction in power stability.

**Figure 4:**
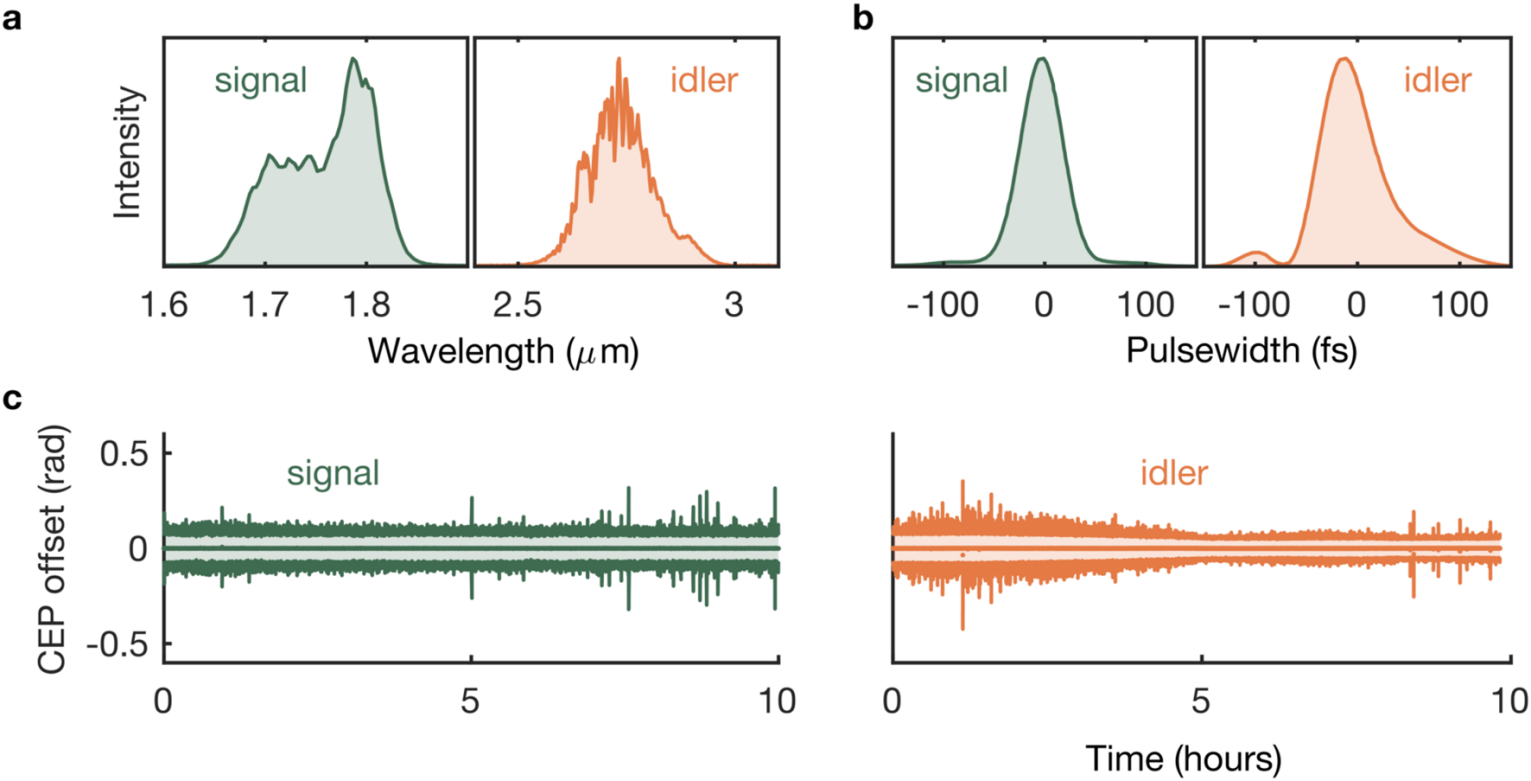
Actively CEP-stabilised HHG modes. (**a**) Spectra of the 1.75-$\upmu$m and the 2.8-$\upmu$m modes. (**b**) Near-transform-limited pulse durations (FWHM) of 49 fs and 62 fs, respectively, measured with an SHG-FROG. (**c**) Active CEP stabilisation using an f–2f interferometer, capable of single-shot detection (at 10 kHz), and the Dazzler to produce global CEP offsets with rms fluctuations of 86 mrad and 73 mrad over nearly 10 h.

**Figure 5:**
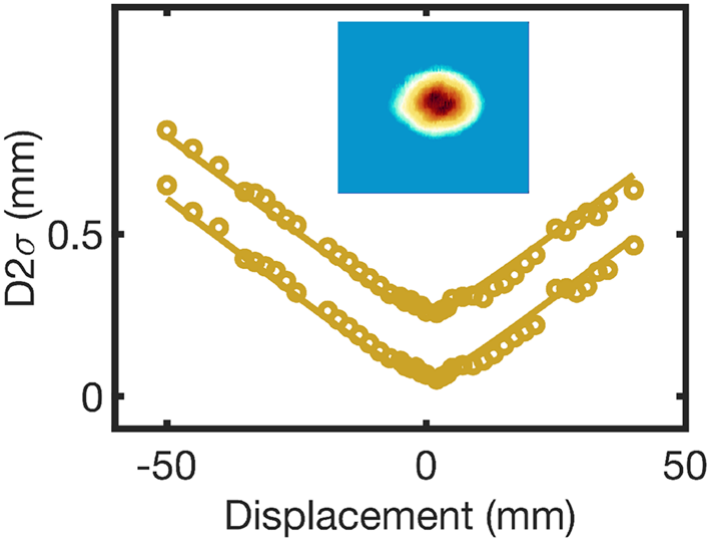
Beam profile. M$^2$ measurement of the HHG-mode signal at 1750 nm, with values of 1.3 and 1.4 along the major and minor axes, respectively. (The plots are vertically offset for clarity). The inset shows the unfocussed beam profile.

The Dazzler-based pulse-shaping can be utilised to generate 19 W of 49-fs signal at 1.75 $\upmu$m and 5 W of 62-fs idler pulses at 2.8 $\upmu$m (Fig. 4a,b). The shorter pulse durations are more amenable to HHG and are achieved using chirped-mirror and bulk-silicon compressors. The long-term power stability is measured to be 1.2% and 2.3% over > 8 h for the broadband 1.75-$\upmu$m and the 2.8-$\upmu$m modes, respectively. Thermal distortions in the propagation of the 2.8-$\upmu$m beam due to water-vapour absorption is minimised by purging the OPCPA with dry air. An $M^2$ measurement (Fig. 5) for the 1.75-$\upmu$m output shows values of 1.3 and 1.4 along the major and minor axes, respectively. 10% of the signal and idler outputs are directed towards two different f–2f interferometers (Fringeezz, Fastlite)^25^, which are capable of single-shot detection (at 10 kHz) and provide active feedback to the Dazzler for CEP stabilisation^26^. The global CEP offset for the 1.75-$\upmu$m and the 2.8-$\upmu$m modes, measured over 10 h, show rms fluctuations of 86 mrad and 73 mrad, respectively (Fig. 4c). Notably, whilst the 2.8-$\upmu$m HHG mode uses the fundamental-driven WLG1 seed, the 1.75-$\upmu$m HHG mode uses a frequency-doubled white-light stage (WLG2) in order to maintain passive CEP stability. This is because the 2.8-$\upmu$m output originates in OPA3, which is pumped at 1030 nm, and is seeded by the amplified light from WLG1, also pumped at 1030 nm. On the contrary, the 1.75-$\upmu$m output originates in the DFG stage, which is pumped at 515 nm. Consequently, for passive CEP stability, it is seeded with WLG2, comprising frequency-doubled white light driven at 1030 nm (since white light driven by a frequency-doubled pump at 515 nm was found to be relatively less stable). The CEP stability will become important following post-compression of the OPCPA output to few-cycle pulses^27^, which is planned for the near future.

## Summary

In summary, we demonstrate the performance of a high-repetition-rate high-average-power four-stage OPCPA, tunable in the infrared with pulse durations ranging from tens to hundreds of femtoseconds, with spectral filtering and active CEP stabilisation. The current OPCPA elevates a previous design^28^ in order to accommodate varying experimental needs, ranging from time-resolved photoelectron to multi-dimensional infrared and Raman spectroscopy. The versatile and multifaceted laser architecture largely depends on the ability to pulse-shape at high repetition rates using the Dazzler. Careful optimisation of the focussing conditions and dry-air purging conditions could generate up to 35 W of uncompressed 1.55-$\upmu$m signal (and 18 W of 3.1-$\upmu$m idler). Since its commissioning, the OPCPA has been successfully employed to generate high-harmonics and perform tr-ARPES experiments. Future efforts will be directed towards exploiting the full potential of the laser in x-ray absorption, 2DIR spectroscopy and Kerr-gated Raman spectroscopy.
